# Survey of dog owners’ and veterinarians’ attitudes regarding the selection of flea and tick prevention products in Thailand

**DOI:** 10.14202/vetworld.2024.1928-1935

**Published:** 2024-08-27

**Authors:** Pornlapas Panchim, Pinrumpai Saengpradub, Sajitha Rakkijpradit, Apichaya Watananontchai, Piyarat Chansiripornchai, Kris Angkanaporn

**Affiliations:** 1 Faculty of Veterinary Science, Chulalongkorn University, Bangkok, 10330, Thailand; 2 Department of Pharmacology, Faculty of Veterinary Science, Chulalongkorn University, Bangkok, 10330, Thailand; 3 Department of Physiology, Faculty of Veterinary Science, Chulalongkorn University, Bangkok, 10330, Thailand

**Keywords:** attitudes, dog, flea, Thailand, tick, veterinarian

## Abstract

**Background and Aim:**

Tick and flea infestations in dogs are significant concerns for both dog owners and veterinarians in Thailand. The country’s climate provides an optimal environment for the proliferation of ticks and fleas, thereby increasing the risk of diseases transmitted by these parasites. At present, a diverse range of tick and flea prevention products is available on the market. This study aimed to investigate the factors influencing the choice of tick and flea prevention products among dog owners and veterinarians in Thailand.

**Materials and Methods:**

Questionnaires were distributed both online and in-person to collect data from dog owners and veterinarians. The data collection period spanned from September 1, 2022, to August 31, 2023. A total of 994 respondents, consisting of 828 dog owners and 166 veterinarians, participated in the questionnaire and were included in this study. The data were then subjected to descriptive statistics and Chi-square test.

**Results:**

The results indicated that most dog owners consider chewable products to be the most effective form of tick and flea prevention, followed by sprays and spot-on treatments. Statistically significant factors (p < 0.05) affecting the frequency of use of tick and flea prevention products were identified, including dog breed, number of owned dogs, and owners’ awareness of tick and flea issues in dogs. Most dog owners tend to use these products when their dogs are infected with ticks or fleas. In addition, owners of a single dog tended to use these products regularly compared to those with multiple dogs. Notably, some dog owners (10.99%) used unregistered products. Among veterinarians, it was observed that dog owners followed the recommendations provided by veterinarians (80.12%). In most cases, veterinarians recommend various tick and flea prevention products (74.10%). The most influential factor affecting product selection is suitability for the individual animal.

**Conclusion:**

This study provides insights for veterinarians and relevant stakeholders regarding the factors and attitudes that influence dog owners’ selection of tick and flea prevention products. This knowledge can contribute to better planning for ectoparasite prevention. In addition, effective communication from veterinarians can increase owner awareness of the importance of tick and flea prevention, thereby reducing the incidence of diseases transmitted by ticks and fleas.

## Introduction

Thailand’s hot and humid climate creates favorable conditions for the proliferation of ectoparasites, such as ticks and fleas, which significantly contribute to subsequent health problems in dogs [1–3]. Diseases commonly found include anemia, skin inflammation, hypersensitivity to flea saliva, and tick-borne illnesses [4]. Ticks and fleas have distinct life cycles; therefore, it is important to select preventive products that specifically target these life cycles. In veterinary medicine, the most significant tick family is the Ixodidae [5]. In Thailand, among the ticks commonly found on dogs, *Rhipicephalus sanguineus*, also known as the brown dog tick, is the most common [3]. This tick has a three-host life cycle consisting of four stages: An egg, a larva (with six legs), a nymph (with eight legs), and an adult. When the eggs hatch into larvae, they seek a host to feed on. Once the larvae have engorged themselves with blood, they detach from the host and molt into nymphs, marking the first host shift. Nymphs feed on a host and then drop off when engorged, undergoing a second host shift before eventually molting into adults. Adult ticks attach to a host once again for feeding, mating, and laying eggs (during the third host shift) [6]. Fleas belong to the order Siphonaptera. The flea species commonly found in dogs, known scientifically as *Ctenocephalides canis*, is commonly referred to as the “dog flea” in common terms. The flea life cycle comprises four stages: Egg, larva, pupa, and adult. In the larval stage, they do not attach to dogs. The larvae form a silk cocoon, known as a pupa, which has a sticky outer surface. Environmental vibrations or movements can trigger the emergence of adult fleas from pupae. Once the flea reaches the adult stage, it jumps back onto the host dog to feed on its blood and reproduce [7]. Understanding the life cycles of fleas and ticks is crucial for effective control and prevention measures in dogs because different stages may require different approaches for eradication [8, 9].

A wide variety of products is currently available for preventing and eliminating ticks and fleas [10]. The effectiveness of these products can vary depending on their type, active ingredients, and application method. Therefore, many dog owners have questions about selecting the most suitable products for their pets, including whether certain products can be used concurrently, the recommended duration of use, and appropriate application methods. Various tick and flea control products are available, including spot-on treatments, chewable tablets, sprays, powders, collars, and shampoos. Commonly used flea and tick control medications include macrocyclic lactones such as selamectin [11], ivermectin, and moxidectin; a derivative of phenylpyrazole (fipronil); gamma-aminobutyric acid receptor antagonists (isoxazolines e.g., afoxolaner, fluralaner, and sarolaner) [12]; as well as imidacloprid, (S)-methoprene, and flumethrin. When recommending tick and flea prevention products, veterinarians can tailor their advice to the dog owners’ preferences and specific needs. These considerations include the product price, frequency of use, age and weight of the pet, and type of product. Providing such guidance can benefit both the dog’s health and the owner’s satisfaction with the protection against tick- and flea-transmitted diseases. Preventing fleas and ticks reduces the risk of diseases for both pets and owners and is considered the most effective approach [7, 10].

Based on the literature review, research conducted in the UK has shown that the key factors influencing pet owners’ choice of flea and tick prevention products are product effectiveness, veterinarian recommendations, frequency of use, and price [13]. However, there is limited information and research on these key factors in Thailand. Thus, the aim of this study was to survey the understanding and reasons for selecting flea and tick prevention products for dogs by pet owners and veterinarians in Thailand. Moreover, this study aimed to explore the factors influencing the choice of such products and to gather insights from both pet owners and veterinarians.

## Materials and Methods

### Ethical approval and Informed consent

The ethics committee exempted this study from requiring ethical approval in accordance with the Institutional Animal Care and Use Protocol because it solely involved administering surveys to pet owners and did not subject any live animals to experimentation. However, pet owners were required to sign a consent form to participate in the study. Written consent was obtained from all respondents before the study commencement.

### Study period and location

The data collection period spanned from September 1, 2022, to August 31, 2023. The study population was categorized into two groups: dog owners and veterinarians. The questionnaires were distributed to both dog owners and veterinarians in paper-based and web-based formats and were available through social media platforms, including Facebook groups and Twitter. The questionnaires were also distributed to dog owners visiting the university’s small animal hospital in Bangkok.

### Sample size and power of the study

The required sample size for dog owners was calculated using G*Power version 3.1 (https://download.cnet.com/g-power/). Based on an effect size of 0.15, power of 0.80, and significance level (alpha) of 0.05., the calculation indicated that 760 respondents were required. Similarly, for the group of veterinarians, G*Power suggested a sample size of 143 individuals based on an effect size of 0.30, a power of 0.80, and an alpha of 0.05.

### Data collection

Before distributing the questionnaires, the authors conducted a pilot test by administering them anonymously to 15 dog owners and five veterinarians. This was conducted to assess the clarity and potential ambiguity of the questions and gather feedback on them. Subsequently, adjustments were made to the questions, and it took approximately 10–15 min to complete all questions.

The questionnaire for the dog owners was divided into three sections. The first section consisted of respondents personal information, including a total of six questions (i.e., gender, education, and income). The second section gathered information about dogs, comprising 11 questions on the number of dogs in the household, breeds of dogs, and activities of dogs. Furthermore, canine breeds were classified into three size categories: small breeds (0–25 lbs), medium and mixed breeds (26–50 lbs), and large breeds (>over 50 lbs), using average weight data reported by the American Kennel Club for each breed [14]. The final section aimed to gather information about the respondents’ experiences with antiparasitic treatments, including 13 questions on the types of products used and the frequency of their use.

The questionnaires designed for veterinarians were divided into two sections: The first section included information about veterinarians (e.g., gender, age, and place of residence), while the second section of the questionnaire focused on the reasons for using tick and flea prevention products.

### Inclusion criteria

The survey established two sets of inclusion criteria, one for dog owners and another for veterinarians. For the dog owner segment, the following inclusion criteria were applied: Ownership of at least one dog, residence in Thailand, and previous use of tick and flea prevention products for their dog. Veterinarians were required to be currently practicing in Thailand.

### Exclusion criteria

The exclusion criteria for respondents included dog owners and veterinarians who provided incomplete responses and those who were not residents of Thailand. Furthermore, dog owners who lacked experience in raising at least one dog or using tick and flea prevention products were ineligible to participate in the study.

### Statistical analysis

Descriptive statistics and the Chi-square test were performed using Statistical Package for the Social Sciences^®^ version 29 for Mac (IBM Corp., NY, USA). To summarize the data, descriptive analysis was conducted, and the frequency distribution of each variable was presented as a percentage. The Chi-square test was used to compare the proportions of information collected from dog owners regarding their attitudes toward tick and flea prevention products. p < 0.05 was considered significant. The satisfaction scores of the dog owners were calculated using weighted averages.

## Results

### Description of the questionnaire items

#### Part 1: Dog owners

Initially, 900 owners responded to the questionnaire, with each participant completing all the questions. However, datasets from 72 respondents were excluded from the study; 6 because they were not dog owners and n = 66 because of a lack of experience using tick and flea prevention products. Therefore, 828 responses were included in the dataset for statistical analysis (Table-1).

**Table-1:** Description of dog owners who responded to a questionnaire investigating attitudes regarding the selection of flea and tick prevention products in Thailand conducted from September 2022 to August 2023 (n=828).

Variable	Category	Total number of respondents (%)
Sex	Male	160 (19.3)
	Female	650 (78.5)
	Others	18 (2.2)
Age (years)	<20	57 (6.9)
	21–30	287 (34.7)
	31–40	187 (22.6)
	41–50	146 (17.6)
	>50	151 (18.2)
Highest level of education	Junior high school	17 (2.1)
	Senior high school or Vocational certificate	49 (5.9)
	Diploma or Associate’s degree	21 (2.5)
	Bachelor’s degree	538 (65.0)
	Higher than bachelor’s degree	203 (24.5)
Income per month (baht)	<10,000	135 (16.3)
	10,001–20,000	150 (18.1)
	20,001–30,000	144 (17.4)
	30,001–40,000	99 (12.0)
	>40,000	300 (36.2)
Resident region	Bangkok and metropolitan areas	741 (89.5)
	Other provinces	87 (10.5)
Experience with raising dogs	<6 months	10 (1.2)
	6–12 months	24 (2.9)
	1–5 years	103 (12.4)
	>5 years	691 (83.5)

Table-1 presents the descriptive characteristics of the respondents. The majority of respondents were female (78.5%), aged 21–30 (34.7%), had a Bachelor’s degree (65%), had a monthly income exceeding 40,000 baht (1,120 USD) (36.2%), and resided in Bangkok and metropolitan areas (89.5%). Furthermore, 83.5% of respondents reported having more than 5 years of experience in raising dogs.

In the dog information section, most respondents indicated that they were raising only a single dog (43.4%), followed by those raising 2 dogs (25.9%). Among the respondents, 52.9% owned medium-sized or Thai-breed dogs, followed by toy- or small-breed dogs (32.4%) and large-breed dogs (14.7%). In addition, most dog owners were raising a dog alongside other dogs (36.2%), followed by those raising only 1 dog (30.1%) and those raising their dog with other types of pets (20.0%). The owners reported that they kept their dogs exclusively indoors, outdoors, and in a mixed environment at rates of 68.2%, 8.0%, and 23.8%, respectively.

**Figure-1:**
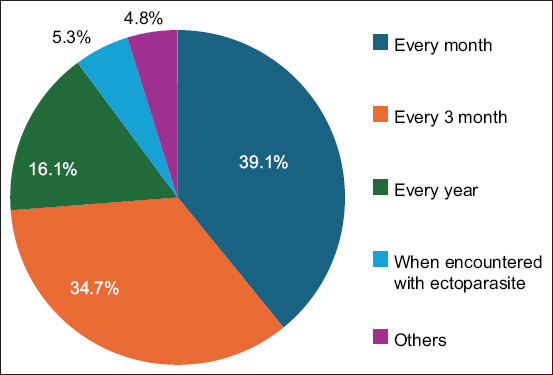
Percentage usage of tick and flea prevention products by dog owners.

In the section concerning the use of antiparasitic products, most owners (77.3%) reported encountering ectoparasite issues. Furthermore, they reported purchasing tick and flea prevention products from various sources (42.8%), had multiple reasons influencing their choice of product type and brand (47.5% and 42.5%, respectively), and used various types of products (35.5%). The dog owners reported monthly usage of tick and flea prevention product (39.1%), followed by every 3 months (34.7%), and yearly usage (16.1%), as shown in Figure-1. In addition, owners expressed high confidence in the effectiveness of these products in preventing ticks and fleas (76.7%). They demonstrated awareness of the diseases transmitted by ticks and fleas (81.8%) and believed that preventing tick and flea infections was of utmost importance for their dogs (88.2%).

Most dog owners choose to use multiple types of tick and flea prevention products, accounting for approximately 35.6%. Other dog owners preferred specific types of products, which were as follows: Spot-on solutions (25.8%), chewable tablets (15.7%), and topical products such as shampoos and powders (9.4%). The injections were relatively less common, constituting only 0.8% of the preferences. Simultaneously, 91 individuals (11%) were found using unregistered tick and flea prevention products, and 16 dog owners (1.9%) reported using an unknown product brand. The satisfaction levels with various product formats were evaluated by dog owners using a scale ranging from 1 (minimum satisfaction) to 5 (maximum satisfaction). The assessment results are presented in Table-2.

**Table-2:** Weighted mean score of dog owner’ satisfaction for each type of tick- and flea-preventive product.

The type of product	Weighted mean
Spot-on	3.45
Chewable tablet	4.01
Injection	3.83
Collar	2.26
Shampoo	2.60
Powder	2.42

The weighted mean total satisfaction scores for tick and flea prevention products, with a maximum score of 5, indicated that dog owners preferred the following products in descending order: Chewable tablets (4.01), injections (3.83), spot-on solutions (3.45), shampoos (2.60), powders (2.42), and collars (2.26).

The results obtained from the Chi-square analysis (Table-3) revealed statistically significant associations between the frequency of preventive product use and factors such as the number of dogs, type of dog breed, and incidence of ectoparasite infestation. Conversely, owner-related factors, such as the highest level of education, income, and experience in raising dogs, did not demonstrate statistical significance (p > 0.05).

**Table-3:** Association between owner-related factors and the frequency of usages of preventive products.

Factors	p-value
Highest level of education	0.492
Income	0.760
Number of dogs	0.002
The type of breed	0.013
Experience with raising dogs	0.262
Encounter problems with ectoparasites	<0.001

#### Part 2: Veterinarians

Initially, 174 veterinarians participated in the questionnaire; however, eight were excluded due to incomplete responses. Therefore, 166 responses were included in the statistical analysis (Table-4).

**Table-4:** Description of a veterinarian responding to a questionnaire investigating attitudes regarding the selection of flea and tick prevention products in Thailand conducted from September 2022 to August 2023 (n=166).

Variable	Category	Total number of respondents (%)
Sex	Male	42 (25.3)
	Female	122 (73.5)
	Others	2 (1.2)
Age (years)	21–30	118 (71.1)
	31–40	37 (22.3)
	41–50	8 (4.8)
	>50	3 (1.8)
Highest level of education	Bachelor’s degree	146 (88.0)
	Master’s degree	13 (7.8)
	Doctoral degree	7 (4.2)
Working region	Bangkok and metropolitan	132 (79.5)
	Other provinces	34 (20.5)
Working experience (years)	<1	47 (28.3)
	1–5	77 (46.4)
	6–10	20 (12.0)
	>10	22 (13.3)

Table-4 presents the descriptive characteristics of the veterinarian respondents. Most veterinarians were female (73.5%), aged 21–30 (71.1%), and had a Bachelor’s degree (88%). Furthermore, 79.5% of the respondents worked in the Bangkok metropolitan area, with work experience ranging from 1 to 5 years (46.4%).

Based on veterinarians’ experience, most pet owners frequently used tick prevention products recommended by veterinarians (80.12%). Furthermore, when veterinarians identified that owners were using inappropriate products, they primarily recommended alternative products better suited for dogs (93.7%).

Regarding ectoparasite prevention, approximately 74 veterinarians employed a combination of products to control ticks and fleas. Furthermore, the weighted mean perception scores among veterinarians regarding pet owners’ knowledge and cooperation in tick prevention measures, with a maximum score of 5, were as follows: Pet owners’ knowledge about tick prevention received a rating of 2.98/5, the level of owner cooperation was rated at 3.48/5, and pet owners’ awareness about diseases transmitted by ticks was rated at 2.58/5. The factors influencing veterinarians were as follows (Table-5) ranked in order of significance: Suitability for the pet (4.29), species of ectoparasite encountered (4.07), types of active ingredients (4.05), familiarity with the product (3.99), duration of efficacy (3.95), pricing and promotional strategies (3.61), and owner preferences (3.44).

**Table-5:** Factors influencing decision-making in selecting tick and flea prevention products in veterinary practice.

Factors influencing the veterinarian’s decision	Weighted mean
Types of active ingredients	4.05
Duration of efficacy	3.95
Species of ectoparasites	4.07
Pricing and promotional strategies	3.61
Suitability of pets	4.29
Familiarity with the product	3.99
Owner’s preferences	3.44

Table-6 demonstrates the factors influencing a veterinarian’s decision, ranked in order of significance: suitability for the pet (4.29), species of ectoparasite encountered (4.07), types of active ingredients (4.05), familiarity with the product (3.99), duration of efficacy (3.95), pricing and promotional strategies (3.61), and owner’s preferences (3.44).

**Table-6:** Factors influencing decision-making in selecting tick and flea prevention products in veterinary practice.

Factors influencing the veterinarian’s decision	Weighted mean
Suitability of pets	4.29
Species of ectoparasites	4.07
Types of active ingredients	4.05
Familiarity with the product	3.99
Duration of efficacy	3.95
Pricing and promotional strategies	3.61
Owner’s preferences	3.44

## Discussion

The aim of this study was to identify the factors influencing pet owners’ and veterinarians’ choice of flea and tick prevention products for dogs in Thailand.

A significant proportion of dog owners preferred monthly product applications over quarterly applications. In contrast, a study in the US showed that dog owners prefer 12-week ectoparasiticides over monthly ones [15]. There was a slight variation in the number of owners choosing a 3-month frequency compared to those choosing a monthly frequency. In this study, we describe a strong association between the frequency of product use and ectoparasite infestation in two ways: First, this finding suggests that dog owners are more likely to use tick and flea prevention products when ectoparasite infestations are detected, reflecting increased awareness among dog owners about tick- and flea-borne diseases. A prior study conducted in Hong Kong similarly revealed that most dog owners were aware of ticks and tick-borne diseases [16]. Second, despite using the products every 3 months and every month as recommended, dogs continue to suffer from ticks and fleas. It is possible that owners may be administering incorrect doses or using unregistered products, which could result in adverse effects for their dogs. Our study found that approximately 10.99% of dog owners reported using unregistered tick and flea prevention products. These findings may reflect the level of knowledge and awareness among dog owners regarding the purchase of these products. The effectiveness of medications depends on the prescribed regimen; inappropriate dosages can lead to treatment failure [15].

This study observed that small dog breeds were associated with a statistically significantly higher frequency of inappropriate products over intervals exceeding 1 year compared with larger dog breeds. This phenomenon may be attributed to the fact that small dog breeds are predominantly housed indoors, resulting in a lower incidence of tick infestations compared to dogs that spend more time outdoors. These findings agree with previous research conducted in Italy [17] and Taiwan [18], which revealed that the factors influencing tick infestations in dogs are closely related to the activities of dogs. Specifically, active dogs outdoors tend to have a higher tick burden than indoor dogs.

Large dog breeds are more likely to encounter ticks than small dog breeds, with a 2.8-fold greater likelihood, possibly because detecting ticks in smaller dogs may be more challenging [19]. This study also revealed that dogs with a history of tick-related issues tend to use appropriate tick-prevention products more frequently. Owners of small dog breeds may encounter difficulties when detecting ticks on their pets. Furthermore, they may not engage in outdoor activities with their dogs as often, leading to less frequent use of tick prevention products.

The association between the number of dogs in a household and the frequency of using ectoparasite prevention measures showed that households with a single dog used antiparasitic drugs more frequently than those with multiple dogs. From the results of this study, it is evident that raising some dogs requires more financial resources for their care and expenses than raising a larger number of dogs. This is because ectoparasite prevention products are expensive, leading to significant costs. In addition, there are other expenses, such as annual vaccinations, healthcare costs, and grooming. Owners may be unable to bear all these expenses, leading to a reduction in the frequency of using tick and flea prevention products or not using them when they have multiple dogs.

Consequently, this may lead to confusion about the best time to apply the product, resulting in its suboptimal use. This study aligns with previous findings by Lue *et al*. [20] that owners with multiple pets are price sensitive (19%), are more concerned about pricing, and might not always be able to afford comprehensive veterinary care. In contrast, price-aware owners (81%) were more willing to spend to ensure the well-being of their pets and placed higher priority on veterinary care.

Most dog owners believe that chewable tablets are the most effective products for tick and flea prevention, followed by injections, spot-on solutions, shampoos, powders, and collars. Our findings align with those of a previous study by Tinkruejeen *et al*. [21] that compared the effectiveness of dioxolane, a chewable tablet, and ivermectin, an injectable medication, in dogs naturally infested with *R. sanguineus*. Afoxolaner (2.7–6.9 mg/kg) exhibited more than 96% efficacy at 24-h post-treatment, and this level consistently remained above 99% from day 14 throughout the duration of the trial. Conversely, monthly subcutaneous injections of ivermectin (at doses of 300 μg/kg) did not effectively control infestations [21]. Moreover, fluralaner (25–56 mg/kg), an example of a chewable tablet, is systemically distributed after oral administration. The study demonstrated that once orally administered, fipronil was highly effective for 12 weeks of tick and flea prevention and was 3 times more effective than topical fipronil (≥6.7 mg/kg), which is typically administered monthly [22, 23].

In addition to efficacy, several factors may influence these owners’ preferences, including recommendations from veterinary professionals, convenience, advice from peers, pricing considerations, and promotional activities. Some factors were similar to those found in research conducted in the UK [13], including effectiveness, recommendations from veterinarians, and product price. Injectable and chewable tablet products are commonly prescribed by veterinarians according to the preferences of pet owners, which are influenced by veterinary recommendations. In addition, this study revealed that both types of products were ranked as the top two choices for dog owners’ satisfaction with each type of tick- and flea-containing product.

In this study, most veterinarians perceived that dog owners generally possess a moderate level of knowledge (2.98/5) regarding ectoparasite prevention and are aware of diseases (2.58/5) that can be transmitted by ticks and fleas. Conversely, veterinarians also believed that most dog owners generally cooperated well (3.48/5) in adhering to their recommendations for ectoparasite prevention. This result is in contrast with the findings of a previous study by Boost *et al*. [16], which reported that most dog owners were aware of the use of tick prevention products. In addition, half of the dog owners were aware of diseases that can be transmitted by ticks, and only a small fraction of them did not use tick-prevention products.

This study revealed that the factors most influential in the decision-making process for selecting tick and flea prevention products among veterinarians are their suitability for dogs. Before prescribing any ectoparasiticide products, a comprehensive assessment of the individual dog’s condition should be conducted to determine which active ingredients can be safely used. The US Food and Drug Administration issued a warning about potential neurological side effects in dogs treated with drugs from the isoxazoline class, including afozolaner, fluralaner, sarolaner, and lotilaner. Isoxazoline products are associated with adverse neurological reactions in some dogs, including muscle tremors, ataxia, and seizures. It is recommended that isoxazoline prescriptions be based on individual patient medical histories [24]. A recent study in the USA revealed a high incidence of neurological adverse effects in dogs treated with isoxazoline, including seizures, ataxia, and trembling, particularly with the use of afozolaner, fluralaner, and sarolaner [25]. From this study, we determined that veterinarians perceive most dog owners to have a moderate level of knowledge about tick and flea prevention. Therefore, owners should consult with a veterinarian before selecting tick and flea prevention products. The suitability of dogs can also influence the types of products veterinarians may choose, considering factors such as age, body weight, and individual health status. Active ingredients from the isoxazoline class are commonly found in chewable tablets. To minimize the risk of adverse neurological events, alternative active ingredients from different classes, which are available in other products, should be considered. In addition, owners should select products based on veterinary recommendations and acquire them from reputable sources.

Most dog owners followed the veterinarian-recommended guidelines for the use of preventive products. A previous study by Adams *et al*. [26] and Maddison [27] suggested that establishing a strong owner-veterinarian bond could occur during the medical interview, particularly when veterinarians attentively listened to the owners’ concerns. Success in preventing tick and flea infestations depends not only on recommendations and reinforcement by the veterinary team but also on the awareness and compliance of pet owners with preventive products. Furthermore, our study indicated that when veterinarians identified that owners were using inappropriate products, they predominantly suggested alternative products better suited for dogs. A previous study by Janke *et al*. [28] found that many veterinarians offer multiple alternative options to their clients rather than a single option to ensure that clients do not feel pressured.

Ectoparasites pose significant risks to the health and welfare of dogs; thus, controlling parasitic infections is a crucial aspect of preventive healthcare. Proper management practices, including the use of effective and safe ectoparasiticides, are required to control these infections. According to our study, dog owners consistently adhere to veterinarian recommendations when selecting ectoparasiticides. Thus, veterinarians should provide evidence to support their decisions, ensuring they are based on rational and responsible drug use. In addition, veterinarians should educate dog owners about the importance of using ectoparasiticides to effectively reduce diseases transmitted by ticks and fleas.

## Conclusion

This study revealed that most dog owners use tick and flea prevention products monthly. Factors contributing to decreased frequency were strongly associated with owning multiple dogs, larger dog breeds, and the absence of tick and flea problems. Owners typically consider multiple factors when choosing ectoparasite prevention products, including convenience, veterinary recommendations, and advice from others, followed by single reasons, including veterinary recommendations and convenience. Suitability for dogs was the most influential factor for veterinarians when selecting prevention products, followed by the species of ectoparasites encountered and types of active ingredients. To enhance awareness of tick- and flea-related issues, educating dog owners about preventive products is advisable. Veterinarians can play a crucial role by providing comprehensive knowledge of these products and their associated diseases.

### Limitations of the study

This study has some limitations due to its reliance on a questionnaire-based approach. First, the accuracy of the questionnaire data depended on the owners’ perspectives, knowledge, and honesty, which may have introduced respondent bias. Second, the questionnaires did not allow researchers to clarify or gather additional information based on the respondents’ answers, which could lead to incomplete responses or misunderstandings. Third, respondents may not have comprehensively represented all Thai dog owners, as most were from Bangkok and other metropolitan areas. The online respondents were likely individuals with regular internet access, whereas the paper-based respondents were probably from the same geographical area or associated with a specific hospital.

Most veterinarians had similar years of experience and worked primarily in Bangkok and other metropolitan areas. This homogeneity in experience and location potentially influences product preferences compared to those with more varied experience or who work in diverse geographical areas. Consequently, the respondents in this study may not fully represent the diverse population of veterinarians in Thailand. In addition, less experienced veterinarians have frequent access to the internet. Consequently, this study received increased collaboration from the veterinarians when responding to the surveys.

### Future implications

This research has enhanced veterinarians’ understanding of dog owners’ perspectives and has facilitated more effective communication between veterinarians and owners. The current study effectively increased awareness regarding the use of ectoparasite prevention products, emphasizing the importance of prevention over treatment to potentially reduce the transmission of tick- and flea-borne diseases. In addition, understanding the dog’s medical history enabled veterinarians to select medications specifically tailored to the dog’s condition. In addition, veterinarians should inform pet owners about the benefits and drawbacks of ectoparasite prevention products. The study also revealed that owners predominantly choose ingestible forms of ectoparasite prevention products, presenting an opportunity for companies to develop such products. This diversification provided owners with a broader range of options for their dogs, enhancing their ability to choose according to their pets’ specific needs.

## Data Availability

The data that support the findings of this study, for example, questionnaires and forms of products are available from the corresponding author on reasonable request.

## Authors’ Contributions

KA, PP, PS, SR, and AW: Data collection and curation. PP, PS, SR, AW, and KA: Formal analysis. KA and PC: Methodology. KA: Project administration. KA, PP, PS, SR, and AW: Original draft. KA and PC: Supervision and editing. All authors have read, reviewed, and approved the final manuscript.
